# *S*-ethyl ethanethiosulfinate, a derivative of allicin, induces metacaspase-dependent apoptosis through ROS generation in *Penicillium chrysogenum*

**DOI:** 10.1042/BSR20190167

**Published:** 2019-06-14

**Authors:** Feilong Qi, Chen Zhang, Shanshan Jiang, Qian Wang, Kudelaidi Kuerban, Man Luo, Mengxue Dong, Xinguang Zhou, Laiming Wu, Biao Jiang, Li Ye

**Affiliations:** 1 Department of Microbiological & Biochemical Pharmacy, School of Pharmacy, Fudan University, Shanghai 201203, China; 2 Shanghai Institute of Organic Chemistry, Chinese Academy of Sciences, Shanghai, China; 3 Department of Pharmacy, Shanghai Ninth People’s Hospital, Shanghai Jiao Tong University School of Medicine, China; 4 Shanghai Museum Conservation Center, Shanghai, China

**Keywords:** antifungal, apoptosis, metacaspase, reactive oxygen species, S-ethyl ethanethiosulfinate

## Abstract

Allicin can be used as fumigant to protect food and cultural relics from fungal contamination because of its strong antifungal activity and the characteristics of high volatility and no residues. However, the obvious disadvantages such as high minimal inhibitory concentration and instability prevent it from wide application. In this study, a stable derivative of allicin, *S*-ethyl ethanethiosulfinate (ALE), was synthesized. We further explored its antifungal activity and apoptosis-inducing effect, as well as the underlying mechanism. ALE had an excellent capability of inhibiting spore germination and mycelial growth of *Penicillium chrysogenum* observed by inverted microscope and scanning electron microscopy. XTT colorimetric assay indicated ALE could reduce the cell viability obviously and IC_50_ was 0.92 μg/ml, only 1/42 of allicin (38.68 μg/ml). DHR 123 ROS Assay Kit, flow cytometry assay and confocal immunofluorescence revealed intercellular ROS generation and metacaspase-dependent apoptosis triggered by ALE, while antioxidant tocopherol could reverse ALE-induced cytotoxicity effect and metacaspase activation. These results indicate that ALE induces metacaspase-dependent apoptosis through ROS generation, thus possesses an effective antifungal activity*.* This new derivative of allicin might be developed as a high efficient alternative to the conventional fungicides for food storage and cultural relic protection.

## Introduction

Fungal contamination is one of the main problems faced in storage of food and grain as well as in protection of cultural relics [1–3]. However, the wide application of synthetic antimicrobial agents used in grain storage and food preservation has severely harmful effect on human health and environment [4]. Meanwhile, synthetic fungicides are generally used in cultural relic protection, but the damage of historical relics caused by chemical residues is indispensable. Thus, it is extremely urgent to develop safer and more environment-friendly antifungal agents, which could effectively protect food and cultural relics from fungal and leave no residues.

Natural products have increasingly popular applications in medicine and green agriculture due to their mild and largely harmless properties in comparison with chemical agents [5–7]. Allicin (diallyl thiosulfinate) is the primary active ingredient of freshly crushed allium species which has a variety of antimicrobial activities including antifungal activity, antiviral activity and antibacterial activity, and considered as a broad-spectrum antibiotic compound [8–11]. More important, it is extraordinary difficult for microbe to obtain resistance to allicin because of its distinctive chemical structure [12,13]. Allicin inhibits both the germination of spores and the growth of hyphae. All of these advantages, as well as its high volatility, propel this molecule into a prime candidate for grain storage and cultural relic protection. Unfortunately, allicin itself is unstable, and can convert into different structures rather quickly [14]. Besides, its minimal inhibitory concentration (MIC) is relatively higher as compared with conventional antibiotics such as amphotericin B and penicillin, which limits its wide application [15,16].

To overcome these disadvantages, we synthesized a new derivative of allicin, *S*-ethyl ethanethiosulfinate (ALE), while retaining the structure of thiosulfinate which was the active group of allicin. With diallyl being saturated, ALE is more stable than natural allicin, and suitable for large-scale production, transportation and storage. The present study investigated for the first time the cytotoxic effects of ALE on *Penicillium chrysogenum*, with allicin as a reference. The results showed that ALE had significantly enhanced antifungal activity. We also investigated the apoptosis-inducing activity and its underlying mechanism.

## Materials and methods

### Chemistry

All solvents and reagents were used without purification. Reactions were monitored by thin-layer chromatography (TLC, Sinopharm Chemical Reagent Co., Ltd., China) and column chromatography was implemented on silica gel H (10 ± 40 mm, Sinopharm Chemical Reagent Co., Ltd., China). Infrared (IR) spectra were measured using Fourier transform (FT)-IR spectrometry (Avatar-360, Nicolet, U.S.A.) and only major peaks were reported. Nuclear magnetic resonance (NMR) spectra were taken with a Bruker DRX 300 (Bruker, Germany). Liquid chromatography mass spectrometry (LC–MS) analysis was conducted on Agilent Technologies 1200 Series System (Agilent Technologies, U.S.A.) and an Agilent Technologies 6110 Quadrupole LC/MS system (Agilent Technologies, U.S.A.).

### Synthesis of *S*-ethyl ethanesulfinothioate

Diethyl disulfide (24.4 g, 0.2 mol) and a catalytic amount of l-proline (700 mg) in acetonitrile (15 ml) were added drop-wise to hydrogen peroxide (34 g, 30%, 0.3 mol) and churned at room temperature (20–25 °C) for 48 h under the protection of N_2_. After the diethyl disulfide was almost consumed, acetonitrile was moved away under reduced pressure and then the resultant outcome was extracted by using CH_2_Cl_2_ (250 ml × 2), purged with saturated brine (10 ml × 2), then dried overnight with anhydrous Na_2_SO_4_. The product was concentrated under reduced pressure to remove CH_2_Cl_2_ and the yield of ALE was normally 90%. ^1^H NMR (300M, CDCl_3_) δ 1.19–1.32 (m, 6H), 2.92–3.01 (m, 4H); LR-ESI: [M+H]^+^ 139.1, [M+Na]^+^ 161.2. IR (cm^−1^): 3711.5, 2971.5, 2929.0, 1451.2, 1321.3, 1262.2, 1128.6, 1082.2, 967.9, 776.3, 492.5.

### Fungal strain and culture conditions

The fungal strain, *Penicillium chrysogenum*, was acquired from the China General Microbiological Culture Collection Center (CGMCC, No. 3.15725) and stored in PDA (Huankai, Guangdong, China) slant at −4°C. Before test, the spore suspension was obtained by washing strain slant with sterile normal saline and shaken up, then adjusted to 5×10^5^ spores/ml. The strain was cultured in PDB (Huankai, Guangdong, China) at 28°C.

### Growth assay

Five mg/ml of ALE stock solution (dissolving 5 mg of ALE in 10% Tween 80) was diluted with normal saline to different concentrations of 0.75, 3 and 6 μg/ml. Natural allicin (Macklin, Shanghai, China) solutions (60, 120 and 250 μg/ml) were used as positive control, while normal saline as negative control. Spore suspensions were seeded in 96-well plates at 90 μl/well. Each well was treated with 10 μl of ALE solutions, allicin solutions or normal saline mentioned above and cultured for 20 h at 28°C, then morphology of *P. chrysogenum* was observed by an inverted microscope (Teelen, Shanghai, China).

### Scanning electron microscopy

Spore suspensions were inoculated into six-well plates and incubated with 0.5 or 1 μg/ml of ALE for 12 h at 28°C. Cells were harvested and fixed by PDB containing 2% glutaraldehyde for 15 min. Afterwards cells were collected by centrifuge (Eppendorf, Hamburg, Germany) at 1000 ×*** g*** for 10 min, and added 1 ml phosphate-buffered glutaraldehyde for fix overnight. Scanning electron microscopy (FEI, Hillsboro, U.S.A.) was used to observe the influence of ALE on *P. chrysogenum.*

### XTT colorimetric assay

Various concentrations of ALE and allicin solution were prepared before assay. Spore suspensions were inoculated in 96-well plates at 90 μl/well. Cells in each well were separately incubated with 10 μl/well of ALE solution, allicin solution or saline for 20 h at 28°C. XTT Sodium (Aladdin, Shanghai, China) was mixed with PDB preheated at 60°C to form a 6.6 mmol/l working solution. Phenazine methosulfate (Melonepharma, Dalian, China) was dissolved by PBS buffer to make a 220 mmol/l solution. XTT and PMS working solution were well mixed in equal volume immediately before used. The mixture solution was added to broth at 20 μl/well, then incubated for 2 h at 28°C. Thereafter, the optical density (OD) was detected by microplate reader (Thermo Fisher, MA, U.S.A.). The data were processed and statistically analyzed with the Graphpad Prism 5.

### MTT cytotoxicity assay

The cytotoxicity of ALE on mammalian cells was explored by MTT assay. The human bronchial epithelium BEAS-2B cells (ATCC® CRL-9609™, Manassas, VA) were regularly cultured in RPMI-1640 (CORNING) medium with 10% of fetal bovine serum (Gibco) at 37°C in a 5% CO_2_ atmosphere incubator. The cells (4 × 10^3^/well) in 96-well plates were treated with 0, 0.05, 0.1, 0.5, 1.0 and 1.5 μg/ml of ALE for 14 h. Thereafter, the cells were co-incubated with 0.5 mg/ml of MTT (Aladdin, Shanghai, China) for 2 h at 37°C. Then, the supernatant was removed and DMSO (100 μl/well) was added to dissolve formazan. The OD was measured by microplate reader (Thermo Fisher, MA, U.S.A.).

### Detection of intracellular ROS

Intracellular ROS production in *P. chrysogenum* was measured by DHR123 ROS Assay Kit (KeyGen BioTEC, Jiangsu, China), an approved fluorescent probe for cellular ROS determination [17]. Spore suspensions were cultured in incubator for 2 h, Afterwards the cells were co-incubated with 0.5 and 1.0 μg/ml of ALE solution and 10 μM DHR123 for 18 h. Then the *P. chrysogenum* cells were gathered, cleansed twice with 1 × PBS buffer, and re-suspended into PDB. To quantify the generation of ROS in cells, fluorescence intensity of DHR123 was determined with a microplate reader (Tecan, Männedorf, Switzerland) using a 507-nm excitation wavelength and a 529-nm emission wavelength. This experiment was also performed in the presence of the ROS inhibitor tocopherol (Beyotime, Shanghai, China) at concentration of 10 μM for the cells treated with 0.5 and 1.0 μg/ml of ALE.

### Confocal immunofluorescence

Spore suspensions were cultured in six-well plates. After treated with 0.5 μg/ml of ALE for 0, 6, 12, 18 and 24 h, or in combination with 10 μM of tocopherol (TOC) for 18 h, the *P. chrysogenum* cells were collected and cleaned twice using 1× PBS buffer. Then the cell precipitation was re-suspended into 70% (v/v) ethanol and incubated for 10 min at room temperature. Afterward the cells were collected and cleaned with PBS once again. Then cell mass was co-incubated with MitoSOX™ Red (Thermo Fisher, MA, U.S.A.) and Hoechst 33342 (Thermo Fisher, MA, U.S.A.) for 20 min. The MitoSOX™ Red is a kind of fluorescent molecular microprobe designed for the detection of mitochondrial ROS by live-cell imaging [18]. Finally the suspensions were put onto the microscopic slide, and then observed with a fluorescent microscope (Zeiss, Oberkochen, Germany) using a 100 × oil immersion lens. The cells treated with 50 μg/ml of fluconazole for 18 h were used as positive control because it was reported that azole antifungals such as fluconazole could induce ROS in fungi [19] .

### Apoptosis assay

Different concentrations of ALE solution were prepared with normal saline. Spore suspensions were cultured in 6-well plates at 90 μl/well for 2 h and then treated with 0, 0.25, 0.5 or 0.75 μg/ml of ALE solution at 28°C for 12 h. The cells were collected by centrifuge for 10 min at 1000 × ***g***, and cleansed twice with precooled 1×PBS buffer. Fungal cell precipitation was suspended in 500 μl PBS buffer solution. Then, 5 μl annexin V-FITC (BD, New Jersey, U.S.A.) and 5 μl PI (BD, New Jersey, U.S.A.) were mixed, subsequently incubated in normal temperature in darkness for 20 min. The proportion of annexin V-positive cells was assayed using a flow cytometer (BD, New Jersey, U.S.A.).

### Measurement of metacaspase activity

The measurement of metacaspase activity was conducted by the ‘CaspACE, FITC-VAD-FMK In Situ Marker’ (Promega, Madison, U.S.A.) under the guidance of operation instructions [20,21]. In short, the cells cultivated with 0.5 μg/ml of ALE for 18 h were collected and washed in PBS. Then the precipitation was re-suspended into dyeing solution including 10 μM of FITC-VAD-FMK, and then co-incubated at 37°C for 20 min. The cells were cleansed once and re-suspended into PBS after incubation. Then suspension was observed with a fluorescent microscope (Zeiss, Oberkochen, Germany) using a 100 × oil immersion lens. This experiment was also performed in the presence of the antioxidant TOC at concentration of 10 μM.

### Statistical analysis

The GraphPad Prism 5 was employed for the statistical analysis. Data from this study were presented as mean values with standard deviations (SD) and compared using student *t* test. ** and **** represented separately *P* < 0.01 and *P* < 0.0001 in the research.

## Results

### Synthesis of *S*-ethyl ethanethiosulfinate

**Figure 1:**
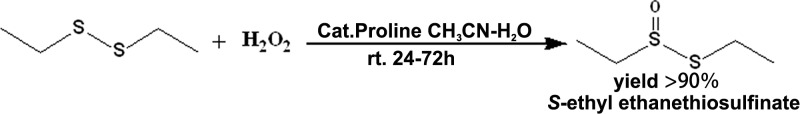
Synthesis pathway of *S*-ethyl ethanethiosulfinate

The synthesis of ALE is depicted in Figure 1. With commercially available diethyl disulfides as starting materials, ALE was prepared in high yields using an environmentally friendly hydrogen peroxide oxidation reaction under the catalytic action of l-proline [22]. Compared with natural allicin, ALE retained the active group thiosulfinate while diallyl was saturated.

### ALE inhibits the spore germination and mycelial growth of *P. chrysogenum*

**Figure 2:**
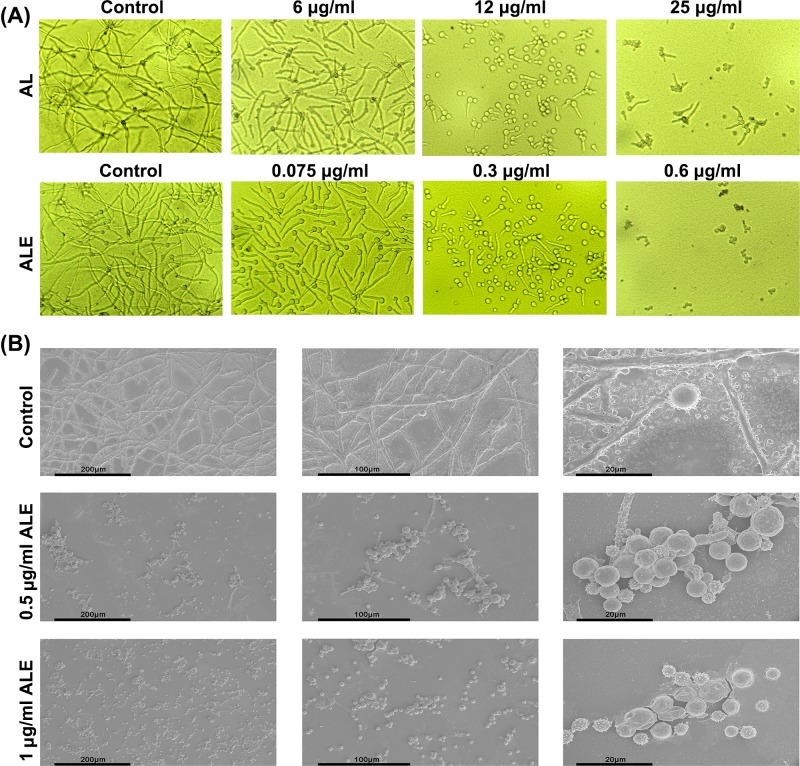
Effect of ALE on spore germination and mycelial growth of *P. chrysogenum* Observation of morphology by inverted microscope (×200) (**A**) and scanning electron microscopy (**B**).

The effect of ALE and natural allicin on spore germination and mycelial growth of *P. chrysogenum* was observed with an inverted microscope. As shown in Figure 2A, both ALE and nature allicin were able to inhibit the spore germination and mycelial growth of *P. chrysogenum* obviously, while mycelia in controls grew well and interlaced. The amount and length of mycelia decreased significantly in the group treated by ALE at dose of 0.075 μg/ml, and spore germination was inhibited obviously after treated with 0.3 μg/ml of ALE. Furthermore, spore shrank and no mycelia were found when the dose of ALE increased to 0.6 μg/ml. In contrast, in order to obtain the same effect, the concentrations of allicin were increased to 6, 12 and 25 μg/ml, respectively. Above all, ALE was more effective and powerful than natural allicin in antifungal activity.

The morphological changes triggered by ALE were traced with SEM (Figure 2B). In control group, mycelia were thick and strong, and spores were spherical and plump. After treated with 0.5 μg/ml of ALE, mycelia hardly grew and spore morphology seemed unchanged. However, spore germination was completely inhibited and spores became irregular and withered when the dosage increased to 1.0 μg/ml. The results further indicated that ALE had an excellent capability of inhibiting spore germination and mycelial growth.

### Cytotoxic effect of ALE on *P. chrysogenum*

**Figure 3:**
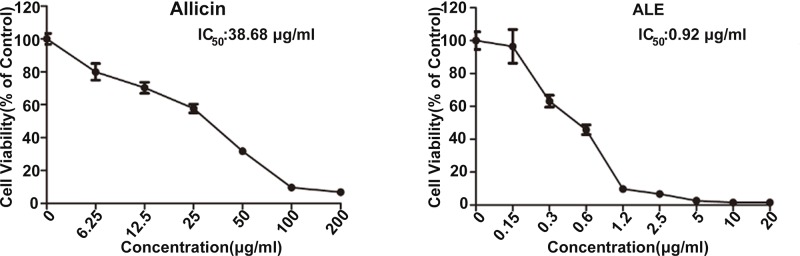
Cytotoxic effect of ALE on *P. chrysogenum* After treated with ALE or allicin, cell viability was determined by XTT colorimetric assay. IC_50_ was obtained by Graphpad Prism 5.

Cell viability was measured through the XTT colorimetric assay to investigate antifungal effect of ALE on *P. chrysogenum*. The data in Figure 3 showed that both ALE and natural allicin reduced cell viability in a dose-dependent manner, while cell viability reduced rapidly especially when the concentration of ALE increased from 0.15 to 1.2 μg/ml. IC_50_ of natural allicin was 36.68 μg/ml, however IC_50_ of ALE was only 0.92 μg/ml. These data indicated that antifungal activity of ALE was more than 42 times as powerful as natural allicin. In addition, ALE had little effect on the viability of human bronchial epithelium BEAS-2B cells, which proved the safety of ALE (Supplementary Figure S1).

### ALE triggers intercellular ROS generation in *P. chrysogenum*

**Figure 4:**
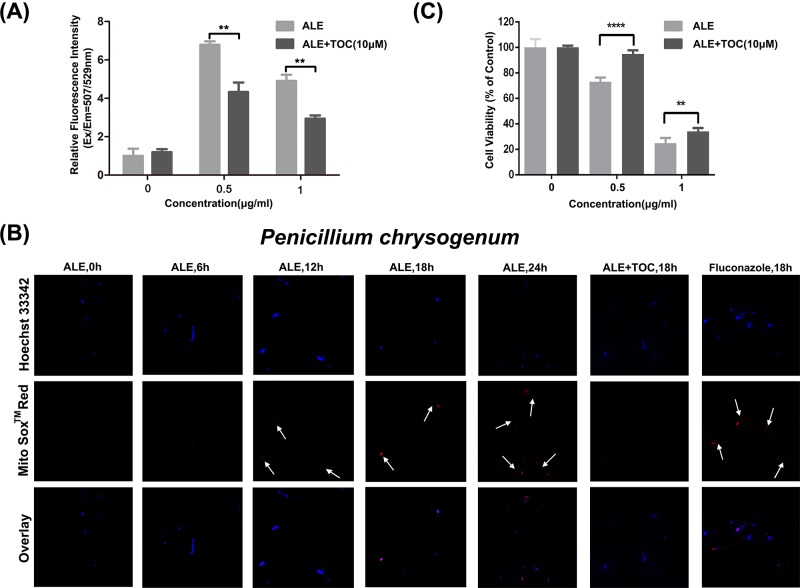
ROS production induced by ALE and its effect on ALE-induced cytotoxicity in *P. chrysogenum* After treated with ALE in absence of presence of TOC, (**A**) intracellular ROS generation was measured by DHR123 ROS Assay Kit. **P < 0.01. (**B**) Fluorescent microscope was used to observe the intracellular ROS generation, with fluconazole as a positive control. (**C**) Cell viability was detected by XTT colorimetric assay. **P < 0.01 and ****P < 0.0001.

We investigated whether ALE induced intercellular ROS generation in *P. chrysogenum*. The production of intracellular ROS could be detected by DHR123. In the existence of ROS, DHR123 can be converted to rhodamine 123 (Rh123) which displays green fluorescence [23]. Spores were incubated with ALE solution in absence or presence of antioxidant tocopherol (TOC), and appraised for the ROS generation according to relative fluorescence intensity. Figure 4A suggested that ALE could induce ROS production markedly in *P. chrysogenum* and the up-regulation was more obvious in group treated with 0.5 μg/ml of ALE, while antioxidant TOC could down-regulate ALE-induced ROS.

We further confirmed intercellular ROS generation using confocal immunofluorescence, with fluconazole as a positive control [19]. MitoSOX™ Red mitochondrial superoxide indicator can be specifically oxidized by ROS and then binds to DNA to give out red fluorescence when illuminated by exciting light of 510 nm [24]. The results in Figure 4B showed that, after 12 h of ALE treatment, cells began to emit very weak red fluorescence. Fluorescence intensity improved tremendously after 18 h treatment and about 50 percent of cells were marked with red. Compared with 18 h, fluorescence intensity of cells treated for 24 h increased obviously, and almost all of cells emitted red fluorescence. These results proved that ALE induced ROS generation in a time-dependent manner. There was not any fluorescence emission in cells co-treated with TOC, indicating that ROS generation promoted by ALE could be removed by TOC.

Cell viability was also detected using XTT colorimetric assay. The results in Figure 4C showed that cell viability was promoted evidently in the existence of TOC, which indicated that clearing ROS by antioxidant TOC could alleviate the cytotoxicity of ALE on *P. chrysogenum.*

Together, these results elucidated that ALE triggered the cytotoxicity effect via intercellular ROS generation in *P. chrysogenum.*

### ALE induces apoptosis in *P. chrysogenum*

**Figure 5:**
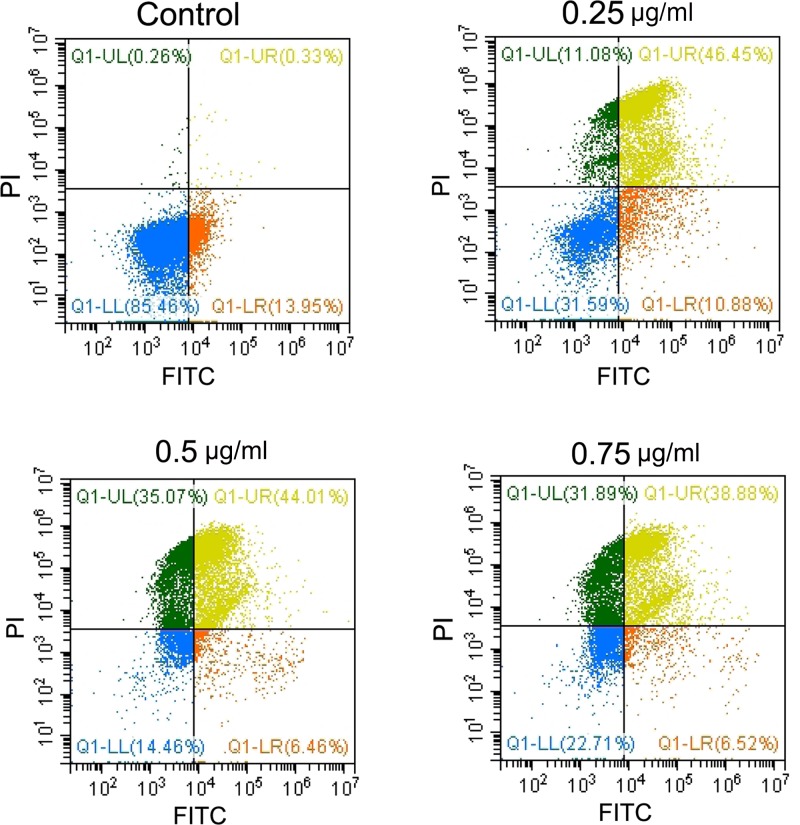
ALE induced apoptosis in *P. chrysogenum* After treated with ALE, cell apoptosis were analyzed by using a flow cytometer.

Flow cytometry assay showed an overall up-regulated trend of the percentage of early and late apoptosis cells with increase in ALE concentration. As shown in Figure 5, total apoptosis rate increased from 14.28% to 57.33% rapidly when the concentration of ALE arrived at 0.25 μg/ml. The result illustrated that ALE was able to induce apoptosis in *P. chrysogenum*.

### ALE-induced apoptosis is metacaspase-dependent and related to ROS

**Figure 6:**
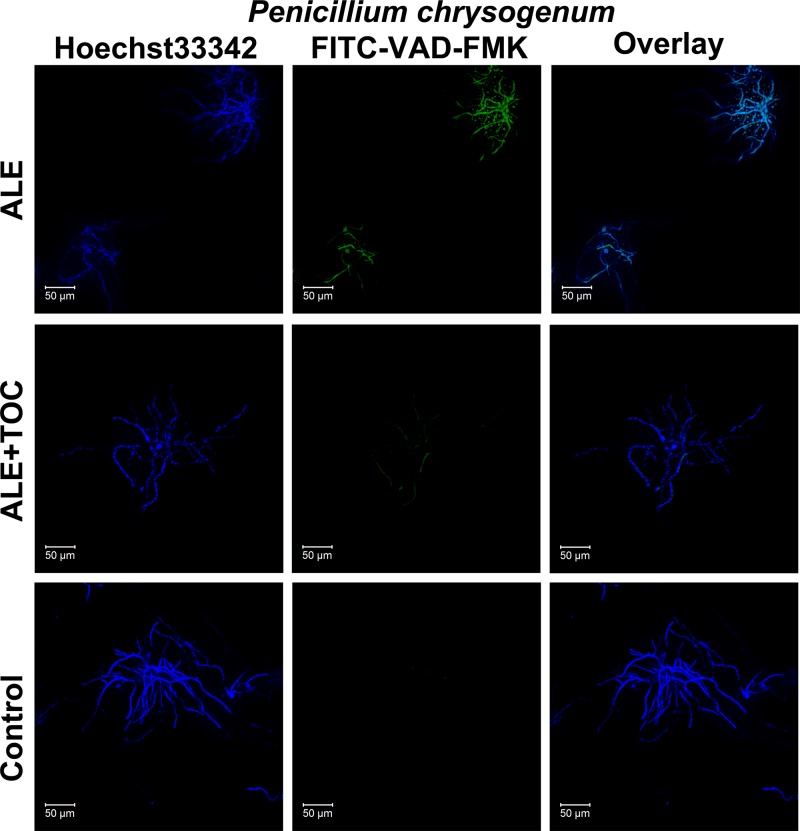
ALE-induced apoptosis was metacaspase-dependent After treated with ALE in absence of presence of TOC, the green fluorescence of metacaspase was observed by using a fluorescent microscope.

Caspase is a series of important proteins related to apoptosis in mammalian cells, but fungal cells only have its analogous and homologous protease, metacaspase [25]. Metacaspase can combine with FITC-VAD-FMK to produce green fluorescence at 493 nm. Observed with a fluorescent microscope, the group exposed to ALE emitted strong green fluorescence indicating activation of metacaspases. However, cells with green fluorescence almost did not exist in the control group (Figure 6). This result indicated that ALE was able to enhance the level of metacaspase activity visibly and the apoptosis triggered by ALE was metacaspase-dependent. Furthermore, activation was dispelled in the presence of TOC because only a few cells shined extremely weak green fluorescence. All the results demonstrated that ALE aroused intercellular ROS generation and then induced metacaspase-dependent apoptosis.

## Discussion

Allicin has become the research hotspot since it was discovered. This kind of main active constituent extracted from garlic played a great role in defense against severe France plague in 1721, which opened the antimicrobial application history of allicin [26]. Curtis et al. found allicin had strong cytotoxicity to fungi which was manifested by the inhibition of the spore germination and mycelial growth no matter *in vivo* and *in vitro* [27]. It had been confirmed that the growth of *Candida albicans, Cryptococcus* and *Aspergillus* were all affected easily by allicin [28–30]. Allicin could destroy membrane potential and induced apoptosis of yeast [31], it also had good synergistic antifungal effect with amphotericin and decreased its MIC obviously [32]. Besides, it was found that allicin could prevent and treat the mold contamination of plant grain, and its performance was similar to common seed disinfectant [33].

The statistics investigated by the United Nations’ Food and Agriculture Organization (FAO) revealed that over 25% of agricultural products worldwide were contaminated by fungi which resulted in huge economic loss [34]. Meanwhile, the contamination and subsequent deterioration of archival materials are also serious threats to cultural relics [35]. The fungi associated with food and archival collections have been identified as species of *Penicillium, Aspergillus, Fusarium*, etc. Because of its wide antimicrobial activity and characteristics of high volatility and no-residue, allicin can be used as fumigant to protect food and cultural relic from fungal contamination. However, the obvious disadvantages of allicin such as high MIC and instability restrict its wide application.

In the present study, we obtained a stable allicin derivative ALE through structural modification, and first explored its antifungal effect. Recently, there were several researches concerning the application of allicin and garlic extracts to prevent pathogenic fungi, and confirmed that allicin and other sulfur compounds in garlic extracts had a significant effect on inhibiting the conidial germination and mycelial growth of *Penicillium* [36,37]. In agreement with these published data, we found ALE could also obviously prevent spore germination along with mycelial growth of *P. chrysogenum.* Moreover, ALE at an extremely low concentration has a stronger efficacy than allicin, displaying an excellent antifungal effect. The observation through scanning electron microscope further proved that ALE could induce deformation of spore and prevent the growth of mycelia. XTT assay showed ALE was able to decrease cell viability of *P. chrysogenum*, and its IC_50_ was less about 42 folds than that of allicin. Above experiments altogether indicate that antifungal effect of ALE is much better than allicin, but the underlying mechanism needs to be further clarified.

Previous published studies about the antimicrobial mechanism of allicin mainly focused on the oxidative inactivation of thiol-containing enzymes via the thiol-disulfide exchange reactions in the microorganisms [38]. Recent researches strongly support the opinion that the oxidative damage induced by intracellular ROS accumulation is a differential action mode of many antifungal agents [39–41]. It has been reported that allicin could enhance the oxidative damage activity of amphotericin B and exert a synergistic fungicidal effect [15]. Measurement of intracellular ROS in this study showed ALE could significantly induce ROS generation in *P. chrysogenum.* Removing ROS by using TOC, an antioxidant, distinctly reversed the damage caused by ALE. These results elucidated that ALE triggered the cytotoxicity effect via intracellular ROS generation.

ROS has been regarded as a significant regulator of cell apoptosis, a kind of programmed cell death [21]. In addition, allicin could induce apoptosis in yeast cells via its oxidization properties [31]. Therefore, we explored whether the ROS accumulation triggered by ALE could initiate apoptosis in *P. chrysogenum*. Flow cytometry assay showed an up-regulated trend in the percentage of apoptosis cells with the increase in ALE dose, illustrating that ALE was able to induce apoptosis in *P. chrysogenum*. It was reported that some antifungal active drugs could induce apoptosis through a metacaspase-dependent pathway [21]. Metacaspases are orthologues of the caspase family identified in fungi and plants, and their activation play a central role in fungal apoptotic-like cell death [42]. The present study detected the activity of metacaspase through CaspACE™ Assay Kit, and the result indicated that ALE indeed activated the metacaspase and induced metacaspase-dependent apoptosis. Meanwhile we found that antioxidant TOC could extraordinarily eliminate the activation of metacaspase induced by ALE, suggesting that it was intracellular ROS accumulation which triggered metacaspase-dependent apoptosis.

**Figure 7:**
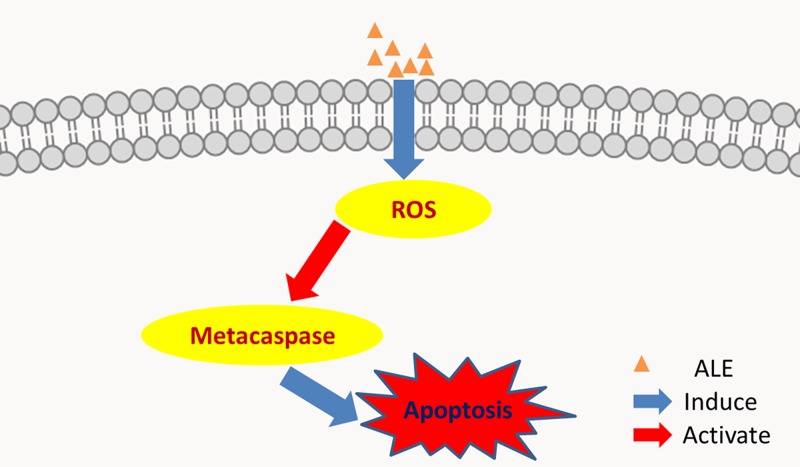
Overview of metacaspase-dependent apoptosis induced by ALE in *P. chrysogenum*

Combined with all the experimental results together, we can obtain the conclusion that ALE induces metacaspase-dependent apoptosis via intracellular ROS generation in *P. chrysogenum*, thus performs an excellent antifungal effect (Figure 7). Therefore, the new allicin derivative ALE might be considered as a potential alternative with high efficiency and volatility to conventional fungicides for food storage and cultural relic protection.

## Abbreviations

ALE: *S*-ethyl ethanethiosulfinate
IR: infrared
LC–MS: liquid chromatography mass spectrometry
MIC: minimal inhibitory concentration
NMR: nuclear magnetic resonance
OD: optical density
